# The complexity, function and applications of RNA in circulation

**DOI:** 10.3389/fgene.2013.00115

**Published:** 2013-06-18

**Authors:** Alton Etheridge, Clarissa P. C. Gomes, Rinaldo W. Pereira, David Galas, Kai Wang

**Affiliations:** ^1^Pacific Northwest Diabetes Research Institute, SeattleWA, USA; ^2^Institute for Systems BiologySeattle, WA, USA; ^3^Graduation on Genomic Sciences and Biotechnology, Catholic University of BrasíliaBrasília, Brazil; ^4^Luxembourg Centre for Systems Biomedicine, University of LuxembourgLuxembourg, Luxembourg

**Keywords:** microRNA, exosomes, microvesicles, cell–cell communication, exogenous RNA

## Abstract

Blood carries a wide array of biomolecules, including nutrients, hormones, and molecules that are secreted by cells for specific biological functions. The recent finding of stable RNA of both endogenous and exogenous origin in circulation raises a number of questions and opens a broad, new field: exploring the origins, functions, and applications of these extracellular RNA molecules. These findings raise many important questions, including: what are the mechanisms of export and cellular uptake, what is the nature and source of their stability, what molecules do they interact with in the blood, and what are the possible biological functions of the circulating RNA? This review summarizes some key recent developments in circulating RNA research and discusses some of the open questions in the field.

Coordination of the activities of different cells in the body is vital for multi-cellular organisms. For this purpose, at least three different types of intercellular communication systems have evolved. Physical interactions between cells through cell surface protein engagement like that between receptors and ligands are used to transmit signals between close, contacting cells (Schonbeck and Libby, 2001; Chillakuri et al., 2012). Gap junctions can also facilitate the transmission of molecular signals between cells (Valiunas et al., 2005; Wolvetang et al., 2007; Lim et al., 2011). Membrane nanotubes or cytonemes that extend up to 100 μm in length are physical connections that also allow communication between cells that are not immediately adjacent to each other (Gerdes and Carvalho, 2008). Multi-cellular organisms have also developed a network of circulating body fluids to deliver nutrients to cells, remove waste molecules, and transmit signals for “long-distance” cell–cell and organ–organ communication. The combination of the circulation system with central and peripheral neural networks, and other short and medium range communication processes, allows cells and organs in an organism to operate in close concert. Some circulating proteins or peptides are well-known for their roles as signaling molecules. These include hormones such as insulin, growth hormone, prolactin, and many other secreted proteins/peptides. In addition to these well-known examples, other biomolecules such as nucleic acids are also exported from cells and are present in various body fluids (Valadi et al., 2007; Weber et al., 2010), although their potential roles in intercellular signaling have not been confirmed. While the discovery of circulating extracellular nucleic acid is not new, having been described as early as 1948 (Mandel and Métais, 1948), there has been a renewed interest in the function and application of circulating nucleic acids due to several, diverse recent findings. These include the discoveries of fetal DNA in maternal blood (Lo and Chiu, 2012), tumor-derived DNA in circulation (Kohler et al., 2011) and stable regulatory non-coding RNA (ncRNA) in body fluids (Cortez et al., 2011).

RNA is generally considered to be an unstable molecule that is subject to degradation and turnover by ubiquitous RNase activity. However, intact RNAs of different types, including both protein-coding RNA (mRNA) and non-coding RNA (ncRNA) have been detected in circulation (Valadi et al., 2007). Some of these RNAs are likely the result of cell lysis through normal cell turnover and are in the process of degradation, reabsorption, or excretion (Turchinovich et al., 2011). However, the abundance and high stability of some of these RNA molecules, especially the short regulatory RNA, microRNA (miRNA), suggests a different scenario: the possibility of a cell–cell communication system through RNA-mediated signals (Lotvall and Valadi, 2007; Turchinovich et al., 2011; Montecalvo et al., 2012). Furthermore, recent studies have shown that exogenous RNA molecules can enter into circulation through the diet or other means, and can potentially be taken up by cells and thereby alter the cellular transcriptome (Semenov et al., 2012; Wang et al., 2012; Zhang et al., 2012a). Many questions remain unanswered, however, including: what are the sources of endogenous and exogenous RNA molecules in circulation, what is (are) the mechanism(s) by which these RNAs enter into circulation, how are they stabilized there, are they taken up by cells, and how are they recognized for uptake by cells? Here we review several recent developments in the study of circulating RNAs and their potential functions and possible applications.

## EXTRACELLULAR RNA IN BODY FLUIDS

Among all the circulating nucleic acids, miRNAs are probably the most extensively studied. These small molecules are short, ncRNAs involved in regulating the cellular transcriptome and proteome by destabilizing mRNA and/or attenuating protein translation (Filipowicz et al., 2008; Fabian et al., 2010). The interaction between miRNAs and their cognate mRNA targets is mediated by partial sequence complementarity between the two (Fabian et al., 2010). The RNA-induced silencing complex (RISC), which contains a number of proteins including members of the Argonaute family, the RNA recognition motif containing protein TNRC6B, putative DNA helicase MOV10, among others, is involved in the miRNA–mRNA interaction (Chendrimada et al., 2007).

MicroRNAs were first detected in the blood plasma and serum in 2008 (Chen et al., 2008; Chim et al., 2008; Hunter et al., 2008; Lawrie et al., 2008; Mitchell et al., 2008; Skog et al., 2008) and have subsequently been detected in many other different body fluids including urine, saliva, tears, and breast milk (Park et al., 2009; Hanke et al., 2010; Weber et al., 2010). The total amount and the concentration of individual miRNAs vary widely among the different fluid types (Weber et al., 2010). The potential of circulating miRNAs as biomarkers has been extensively investigated in recent years. miRNAs make good biomarker candidates for several reasons. They are stable in body fluids, which are easy to obtain from patients, they can be easily measured with high sensitivity because they are amplifiable, and some markers and sets of markers have shown profiles associated with specific pathologies. The changes in miRNA levels may be used as a diagnostic tool for several types of cancer (Mitchell et al., 2008; Taylor and Gercel-Taylor, 2008; Park et al., 2009; Hanke et al., 2010; Huang et al., 2010; Zhao et al., 2010; Moussay et al., 2011; Roth et al., 2011), cardiac damage (Ai et al., 2010; Corsten et al., 2010; Tijsen et al., 2010; Wang et al., 2010a), muscular injury and pathologies (Laterza et al., 2009; Mizuno et al., 2011; Vignier et al., 2013), diabetes (Chen et al., 2008; Erener et al., 2013), liver injury (Laterza et al., 2009; Wang et al., 2009, 2013; Starkey Lewis et al., 2011), among others.

The literature reporting correlations with human disease and pathologies has grown rapidly in the past few years. The number of papers, for example, on changes in circulating miRNAs in cases of myocardial infarction (MI) and cardiac diseases alone has recently exploded. We estimate that more than 30 reports on this specific topic alone have been published since 2009. Some of the circulating miRNAs identified in MI and cardiac diseases have been summarized in **Table 1**.

**Table 1:** Circulating miRNAs with biomarker potential to diagnose acute myocardial infarction (AMI).

Candidate miRNA(s)	Organism	Source	Sample size	Correlation	Reference
1, 133a, 133b, 499-5p	Human; mouse	Plasma	33 patients, 17 healthy; 4–5 AMI and control mice	Elevated levels distinguished cardiac damage patients from healthy subjects. Positive correlation with TnI^a^	D’Alessandra et al. (2010)
1	Human; rat	Serum	31 patients, 20 healthy; 8~AMI rats, 8 controls	High expression was associated with CK-MB^b^; positive correlation with myocardial infarct size	Cheng et al. (2010)
1	Human	Plasma	93 patients, 66 healthy	Up-regulation correlated with QRS^c^ duration	Ai et al. (2010)
208a	Human	Plasma	33 patients, 30 healthy	Present only in patients and showed to be more specific and sensitive than TnI^a^	Wang et al. (2010a)
208b, 499	Human	Plasma	32 patients, 36 healthy	High levels correlated with TnT^d^ and CPK^e^	Corsten et al. (2010)
499	Human	Plasma	9 patients, 10 healthy	Elevated expression correlated positively with CK-MB^b^ activity	Adachi et al. (2010)
1, 133a	Human	Serum	29 patients, 42 non-AMI	Increased levels showed correlation with TnT^d^	Kuwabara et al. (2011)
133, 328	Human	Plasma, whole blood	51 patients, 28 healthy	High levels correlated with TnT^d^	Wang et al. (2011)
30c, 145, 1291, 663b	Human	Whole blood	20 patients, 20 non-AMI	Elevated levels of 30c and 145 correlated with TnT^d^; 1291 and 663b distinguished patients from healthy subjects	Meder et al. (2011)
208b, 499	Human	Plasma	510 patients, 87 healthy	Increased levels correlated with peak concentrations of CPK^e^ and TnT^d^	Devaux et al. (2012)

a Cardiac troponin I;

b creatine kinase muscle b;

c QRS complex in an electrocardiogram;

d cardiac troponin T;

e creatine phosphokinase.

Efforts have also been made to use circulating miRNAs as a prognostic tool in response to therapeutic treatments (Ma et al., 2012). Furthermore, the possibility of using miRNAs as therapeutics themselves has been considered. It is a very exciting prospect if these molecules can be packaged and released into circulation as stable and perhaps even targeted packages. Applications in this area have taken different approaches. For example, creating synthetic miRNAs designed to target specific disease-associated genes, as used by Suckau et al. (2009) to treat heart failure. In this case, viral vectors containing short hairpin RNAs were administered intravenously in rats to reduce the level of a key regulator of cardiac calcium homeostasis, phospholamban (Suckau et al., 2009). The use of antagomirs to target miRNAs that are upregulated in pathological states has also been demonstrated. For example, an antagomir of miR-133 caused sustained cardiac hypertrophy in mice (Care et al., 2007); antagomirs targeting miR-1 and let-7f were successfully used in rats to extend neuroprotection after ischemic stroke (Selvamani et al., 2012); mice treated with high doses of an antagomir for miR-126 showed reduced ischemia-induced angiogenesis (van Solingen et al., 2009); and a miR-206 antagomir increased brain-derived neurotrophic factor levels and improved memory function in rats with Alzheimer’s disease (Lee et al., 2012). Furthermore, a treatment for hepatitis C virus (HCV) chronic infection using an antagomir for the liver-specific miR-122 is currently in clinical trial (Janssen et al., 2013).

## RNA EXPORT, PACKAGING, AND UPTAKE

While extracellular RNAs were first thought to be molecular debris released by cell lysis (Turchinovich et al., 2011), the difference in the spectrum of RNA within cultured cells and the external medium argues strongly for selective export of some fraction of the circulating RNAs (Wang et al., 2010b). It remains unclear how specific RNAs are targeted for export, and if some are simply released with the cytosol through bulk exocytosis. As free RNA molecules are very likely to be degraded outside the cell, it is generally assumed that circulating RNAs are packaged in some form to avoid RNase degradation. Lipid vesicles, such as exosomes or microvesicles are one type of packaging system used by secreted RNAs (Valadi et al., 2007; Camussi et al., 2011). Exosomes, which are approximately 30–100 nm in diameter, are formed by fusion of multivesicular bodies with the plasma membrane. Microvesicles are larger (100 nm–1 μm) and formed by blebbing of the plasma membrane. Both types of vesicles can contain a number of protein and RNA molecules, although the composition varies widely with the origin of the vesicle (Muller, 2012; Huang et al., 2013).

In addition to lipid vesicles, it has been demonstrated that miRNA in the extracellular environment can complex with high-density lipoproteins (HDL) and at least two RNA-binding proteins *in vivo*, Argonaute 1 (AGO1) and Argonaute 2 (AGO2), and with nucleophosmin 1 (NPM1) *in vitro* (Wang et al., 2010b; Arroyo et al., 2011; Turchinovich et al., 2011; Vickers et al., 2011; Turchinovich and Burwinkel, 2012; Wagner et al., 2013). Furthermore, the spectrum of miRNA associated with HDL depends on the health status of an individual (Vickers et al., 2011). However, it is not known if there are other RNA-binding proteins that form similar complexes to protect miRNAs or other types of RNA in circulation.

Besides miRNAs, lipid vesicles also contain a large number of protein-coding RNAs. Roughly 1300 different mRNA transcripts have been identified in lipid vesicles derived from human or mouse cell lines (Valadi et al., 2007). Interestingly, the mRNA composition and abundance in the exosomes differ from that of the original cell, which suggests the contents in the lipid vesicles are selectively packaged rather than included indiscriminately. The levels of circulating miRNAs as well as several mRNAs in circulation have been shown to be associated with stages and types of cancers (Sueoka et al., 2005; Mitchell et al., 2008; Taylor and Gercel-Taylor, 2008; Ohshima et al., 2010; Zhao et al., 2010; Zhang et al., 2012b), pregnancy (Zhong et al., 2008; Rajakumar et al., 2012; Wu et al., 2012; Zhao et al., 2012; Higashijima et al., 2013), and drug-induced liver injury (Miyamoto et al., 2008; Laterza et al., 2009; Wang et al., 2009, 2013; Bala et al., 2012; Su et al., 2012; Okubo et al., 2013).

The idea of using RNA as a mode of intercellular communication is particularly interesting for several reasons. First, RNA can carry information in a simple and efficient way (such as coding for proteins in mRNA), and, second, it can coordinate cellular activity in a fundamental and essential manner (such as microRNA-mediated transcriptome and proteome regulation). It has been demonstrated that cells can export miRNAs, which can be transferred to and are functional in recipient cells (Kosaka et al., 2010; Zhang et al., 2010; Hergenreider et al., 2012). However, it is inherently difficult to test definitively the functioning of an RNA-based communication system *in vivo*. Conditioned media exchange and co-culture experiments have been used to investigate cell–cell communications, and these methods have been adapted to investigate the possibility of RNA uptake by cells (Liu et al., 2010; Vencio et al., 2011; Yang et al., 2011). Some exosome-derived mRNAs have been shown to be functional in recipient cells (Valadi et al., 2007; Ismail et al., 2013). Furthermore, Ratajczak et al. (2006) showed that microvesicles derived from embryonic stem cells can enhance the survival and expansion of mouse hematopoietic progenitor cells, and are enriched in stem cell related transcripts including Oct-4, Rex-1, Nanog, SCL, and GATA-2. Reporter assays have shown that exosome-derived miRNAs can silence transcripts in recipient cells (Lotvall and Valadi, 2007; Valadi et al., 2007; Camussi et al., 2011; Kogure et al., 2011; Mittelbrunn et al., 2011). While evidence suggests that cultured cells can transfer packaged RNA molecules, it is difficult to demonstrate this as a general mechanism *in vivo*. One of the most important remaining biochemical questions is how the RNA in circulation is protected, packaged, and delivered to and recognized by their targets. The current lack of knowledge about these questions makes the study of the biological functions of circulating RNA both important and difficult.

Besides protein-coding RNAs and miRNAs, there are other more abundant types of regulatory RNA molecules, such as large intergenic ncRNAs (lincRNAs) and small nucleolar RNAs (snoRNAs) in extracellular vesicles (Huang et al., 2013). A recent characterization of neuronal exosomes found RNAs derived from repeat sequences, as well as mRNA and several types of small RNAs. While a majority of small RNA was tRNA (90%), other types of small RNAs including miRNA, snoRNA, and small cytoplasmic RNA (scRNA) were also detected (Bellingham et al., 2012). Since both mRNA and miRNA can be taken up and utilized by cells (see earlier paragraph), there is no reason to think that other types of RNA in circulation do not also have functional implications when they are taken up by cells. However, there is currently no direct experimental evidence to support this functional possibility for other types of RNA in circulation.

## METHODS USED IN STUDYING RNA IN CIRCULATION

Microarray, qPCR (quantitative polymerase chain reaction), and sequencing-based approaches are the three major platforms that have been used for miRNA and mRNA profiling. All of these have been widely and successfully applied in assessing the spectra of mRNA and miRNA in cells and tissues. However, due to the relatively low concentration of RNA present in circulation, comprehensive measurement of any circulating RNA is difficult to do accurately and consistently. For miRNAs, the difficulties are compounded by the short lengths of the RNA molecules and the consequent sequence specificity of hybridization free energies. At the moment, the most commonly used method for miRNA profiles is qPCR because of its sensitivity and the limited number of known miRNAs (on the order of 1000 sequences). This method has significant drawbacks as well because of the specificity and differential amplification efficiency of the designed primers. This technique has also been applied to measure the level of specific mRNA sequences in circulation (Wang et al., 2013). Although microarrays have been the gold standard for transcriptome analysis in cells and tissues, the low concentration of RNA in circulation, and the sequence similarity of these short nucleic acid molecules make microarray analysis unsuitable in most cases. The development of next generation sequencing (NGS) has shown promise in measuring circulating RNAs. This platform not only solves some of the miRNA measurement problems related to short and highly similar sequences of miRNAs, but it can provide measurements on a broad spectrum of RNA in a single experiment. Because it does not rely on pre-designed probes or primers, NGS also allows the identification of novel RNA sequences, such as isomirs and exogenous RNAs in circulation (Lee et al., 2010; Wang et al., 2010a, 2012; Semenov et al., 2012). One difficulty with this method is the inherent bias in the preparation of the sequencing libraries. For this reason, absolute measurements of circulating RNA are very difficult, but when carefully done, comparative measurements can be reliable.

## EXOGENOUS RNA IN CIRCULATION

Recent evidence, which we discuss below, suggests that RNAs in circulation are derived not only from cells within the human body, but also from foreign organisms and viruses (Meckes et al., 2010; Pegtel et al., 2010). It has been shown that exogenous RNAs find their way from the outside environment, through diet or from the complex human microbiome (Zhang et al., 2012a). Little is known about how exogenous RNAs are taken up by the gut epithelium from the environment, and there is only very limited and circumstantial evidence so far that these RNAs are functional in the body. However, in keeping with the substantial and growing body of evidence that some human circulating RNA are functional, the possibility that exogenous RNAs, found in the circulation can be taken up and exert specific functions in recipient cells, raises several interesting issues.

A recent report by Zhang et al. (2012a) indicated that exogenous miRNAs ingested from food (rice in this case) could enter into the human circulation and be taken up by and actually function in cells. They found that certain plant miRNAs, including miR-168a, an abundant miRNA in rice, is detectable in human plasma, as well as in plasma from other animals eating a diet containing plant material. However, many questions such as how the RNA survives the cooking process and the harsh environment of the digestive tract, as well as how these biomolecules can pass through the gut epithelium, remain largely unanswered. Using Caco-2 cells transfected with synthetic rice miR-168a, Zhang et al. (2012a) found that transfected miR-168a can be packaged by cells into microvesicles and released into culture medium. Furthermore, their work suggests that the microvesicles containing exogenous miRNA can be taken up by cells and interact with endogenous transcripts both *in vitro* and *in vivo*. Mature miR-168a sequence has significant sequence homology with a liver-expressed transcript, low-density lipoprotein receptor adaptor protein 1 (LDLRAP1) transcript, raising the possibility that it can suppress the expression of LDLRAP1 through the action of the RISC complex. Indeed, mice fed a rice-containing diet have lower level of LDLRAP1 gene expression than mice fed a normal chow diet, suggesting that miR-168a derived from the rice can affect the level of an endogenous mouse transcript (Zhang et al., 2012a).

Recent work by our lab and others have systematically characterized the composition of RNAs in circulation and revealed that some of the circulating RNAs are derived from a variety of exogenous organisms, including the microbiome (Semenov et al., 2012; Wang et al., 2012, 2013). Using a map-and-remove strategy to analyze small RNA-seq data (after removing sequences mapped to human miRNAs, transcripts, and genomic sequences first), reveals that a significant portion of the total reads are of non-human origin. While some of these exogenous RNAs are derived from the diet, the majority of them are of either fungal or bacterial origins (Wang et al., 2012). The sequences detected implicate a wide range of bacterial phyla, including many already known to reside in the gut microbiome. Although there were not significant differences in the plasma RNA spectra between healthy individuals and those with colorectal cancer or ulcerative colitis, differences can be detected in the plasma of people eating different diets, such as a corn-based diet versus a rice-based diet (Wang et al., 2012).

While these results raise the interesting possibility that exogenous RNAs taken up from the environment can alter gene expression in the cells of an organism, many questions remain unanswered. How these RNAs pass through the body’s epithelial lining barrier and enter the circulation, for example, is still completely unclear. Because of the relatively low levels of exogenous circulating RNA, it seems likely that if the packages containing exogenous RNAs exert a significant biological effect on the body’s cells they must be effectively targeted in some way to specific cells or tissues.

Since some miRNAs are highly conserved in metazoans (Shi et al., 2012), the finding of diet derived exogenous RNA in circulation raises the interesting possibility that there are RNA-based processes that either induce or avert the development of diseases in humans arising from foreign RNA. For example, changing the levels of certain highly conserved miRNAs, such as miR-21, which has solid pro-proliferation activity (Sayed et al., 2008; Yao et al., 2009), and miR-150 and miR-146, which have strong association with inflammation activities (Sheedy and O’Neill, 2008; Sonkoly et al., 2008; Schmidt et al., 2009; Quinn and O’Neill, 2011; Zhong et al., 2012), by uptake of exogenous RNAs with similar activities may affect the initiation and progression of certain diseases.

Although promising both as biomarkers and potential functional impacts, such as for therapeutics, there is still a great deal to be learned about RNA in circulation. This newly recognized class of circulating molecules has the potential to be deeply involved in the symbiotic functioning of the human body and its microbiome. We currently know very little about the potential mechanisms of entry of RNAs into circulation, their mechanisms of action, their packaging and export process, and their targeting and uptake by cells. Technical improvements and standardization in measuring the levels of these RNAs as well as new model experimental systems are needed in order to explore the many facets of the transport, targeting, and function of these RNAs. In summary, the health impact of RNAs in circulation seems likely to be significant, but the field is just beginning to be explored and the investigation of the area of the quantitative characterization and specific biological functions of exogenous RNA is even earlier in its development.

## Conflict of Interest Statement

The authors declare that the research was conducted in the absence of any commercial or financial relationships that could be construed as a potential conflict of interest.

## References

[B1] Adachi T. Nakanishi M. Otsuka Y. Nishimura K. Hirokawa G. Goto Y. (2010). Plasma microRNA 499 as a biomarker of acute myocardial infarction. *Clin. Chem.* 56 1183–1185 10.1373/clinchem.2010.144121 20395621

[B2] Ai J. Zhang R. Li Y. Pu J. Lu Y. Jiao J. (2010). Circulating microRNA-1 as a potential novel biomarker for acute myocardial infarction. *Biochem. Biophys. Res. Commun.* 391 73–77 10.1016/j.bbrc.2009.11.005 19896465

[B3] Arroyo J. D. Chevillet J. R. Kroh E. M. Ruf I. K. Pritchard C. C. Gibson D. F. (2011). Argonaute2 complexes carry a population of circulating microRNAs independent of vesicles in human plasma. *Proc. Natl. Acad. Sci. U.S.A.* 108 5003–5008 10.1073/pnas.1019055108 21383194 PMC3064324

[B4] Bala S. Petrasek J. Mundkur S. Catalano D. Levin I. Ward J. (2012). Circulating microRNAs in exosomes indicate hepatocyte injury and inflammation in alcoholic, drug-induced, and inflammatory liver diseases. *Hepatology* 56 1946–57 10.1002/hep.25873 22684891 PMC3486954

[B5] Bellingham S. A. Coleman B. M. Hill A. F. (2012). Small RNA deep sequencing reveals a distinct miRNA signature released in exosomes from prion-infected neuronal cells. *Nucleic Acids Res.* 40 10937–10949 10.1093/nar/gks832 22965126 PMC3505968

[B6] Camussi G. Deregibus M. C. Bruno S. Grange C. Fonsato V. Tetta C. (2011). Exosome/microvesicle-mediated epigenetic reprogramming of cells. *Am. J. Cancer Res.* 1 98–110 10.1093/hmg/dds317 21969178 PMC3180104

[B7] Care A. Catalucci D. Felicetti F. Bonci D. Addario A. Gallo P. (2007). MicroRNA-133 controls cardiac hypertrophy. *Nat. Med.* 13 613–618 10.1038/nm1582 17468766

[B8] Chen X. Ba Y. Ma L. Cai X. Yin Y. Wang K. (2008). Characterization of microRNAs in serum: a novel class of biomarkers for diagnosis of cancer and other diseases. *Cell Res.* 18 997–1006 10.1038/cr.2008.282 18766170

[B9] Chendrimada T. P. Finn K. J. Ji X. Baillat D. Gregory R. I. Liebhaber S. A. (2007). MicroRNA silencing through RISC recruitment of eIF6. *Nature* 447 823–828 10.1038/nature05841 17507929

[B10] Cheng Y. Tan N. Yang J. Liu X. Cao X. He P. (2010). A translational study of circulating cell-free microRNA-1 in acute myocardial infarction. *Clin. Sci. (Lond.)* 119 87–95 10.1042/CS20090645 20218970 PMC3593815

[B11] Chillakuri C. R. Sheppard D. Lea S. M. Handford P. A. (2012). Notch receptor–ligand binding and activation: insights from molecular studies. *Semin. Cell Dev. Biol.* 23 421–428 10.1016/j.semcdb.2012.01.009 22326375 PMC3415683

[B12] Chim S. S. Shing T. K. Hung E. C. Leung T. Y. Lau T. K. Chiu R. W. (2008). Detection and characterization of placental microRNAs in maternal plasma. *Clin. Chem.* 54 482–90 10.1373/clinchem.2007.097972 18218722

[B13] Corsten M. F. Dennert R. Jochems S. Kuznetsova T. Devaux Y. Hofstra L. (2010). Circulating microRNA-208b and microRNA-499 reflect myocardial damage in cardiovascular disease. *Circ. Cardiovasc. Genet.* 3 499–506 10.1161/CIRCGENETICS.110.957415 20921333

[B14] Cortez M. A. Bueso-ramos C. Ferdin J. Lopez-berestein G. Sood A. K. Calin G. A. (2011). MicroRNAs in body fluids – the mix of hormones and biomarkers. *Nat. Rev. Clin. Oncol.* 8 467–477 10.1038/nrclinonc.2011.76 21647195 PMC3423224

[B15] D’Alessandra Y. Devanna P. Limana F. Straino S. Di carlo A. Brambilla P. G. (2010). Circulating microRNAs are new and sensitive biomarkers of myocardial infarction. *Eur. Heart J.* 31 2765–2773 10.1093/eurheartj/ehq221 20534597 PMC2980809

[B16] Devaux Y. Vausort M. Goretti E. Nazarov P. V. Azuaje F. Gilson G. (2012). Use of circulating microRNAs to diagnose acute myocardial infarction. *Clin. Chem.* 58 559–567 10.1373/clinchem.2011.173823 22252325

[B17] Erener S. Mojibian M. Fox J. K. Denroche H. C. Kieffer T. J. (2013). Circulating miR-375 as a biomarker of beta-cell death and diabetes in mice. *Endocrinology* 154 603–608 10.1210/en.2012-1744 23321698

[B18] Fabian M. R. Sonenberg N. Filipowicz W. (2010). Regulation of mRNA translation and stability by microRNAs. *Annu. Rev. Biochem.* 79 351–379 10.1146/annurev-biochem-060308-103103 20533884

[B19] Filipowicz W. Bhattacharyya S. N. Sonenberg N. (2008). Mechanisms of post-transcriptional regulation by microRNAs: are the answers in sight? *Nat. Rev. Genet.* 9 102–114 10.1038/nrg2290 18197166

[B20] Gerdes H. H. Carvalho R. N. (2008). Intercellular transfer mediated by tunneling nanotubes. *Curr. Opin. Cell Biol.* 20 470–475 10.1016/j.ceb.2008.03.005 18456488

[B21] Hanke M. Hoefig K. Merz H. Feller A. C. Kausch I. Jocham D. (2010). A robust methodology to study urine microRNA as tumor marker: microRNA-126 and microRNA-182 are related to urinary bladder cancer. *Urol. Oncol.* 28 655–661 10.1016/j.urolonc.2009.01.027 19375957

[B22] Hergenreider E. Heydt S. Treguer K. Boettger T. Horrevoets A. J. Zeiher A. M. (2012). Atheroprotective communication between endothelial cells and smooth muscle cells through miRNAs. *Nat. Cell Biol.* 14 249–256 10.1038/ncb2441 22327366

[B23] Higashijima A. Miura K. Mishima H. Kinoshita A. Jo O. Abe S. (2013). Characterization of placenta-specific microRNAs in fetal growth restriction pregnancy. *Prenat. Diagn.* 33 214–222 10.1002/pd.4045 23354729

[B24] Huang X. Yuan T. Tschannen M. Sun Z. Jacob H. Du M. (2013). Characterization of human plasma-derived exosomal RNAs by deep sequencing. *BMC Genomics* 14: 319 10.1186/1471-2164-14-319 PMC3653748 23663360

[B25] Huang Z. Huang D. Ni S. Peng Z. Sheng W. Du X. (2010). Plasma microRNAs are promising novel biomarkers for early detection of colorectal cancer. *Int. J. Cancer* 127 118–126 10.1002/ijc.25007 19876917

[B26] Hunter M. P. Ismail N. Zhang X. Aguda B. D. Lee E. J. Yu L. (2008). Detection of microRNA expression in human peripheral blood microvesicles. *PLoS ONE* 3: e3694 10.1371/journal.pone.0003694 PMC2577891 19002258

[B27] Ismail N. Wang Y. Dakhlallah D. Moldovan L. Agarwal K. Batte K. (2013). Macrophage microvesicles induce macrophage differentiation and miR-223 transfer. *Blood* 121 984–995 10.1182/blood-2011-08-374793 23144169 PMC3567345

[B28] Janssen H. L. Reesink H. W. Lawitz E. J. Zeuzem S. Rodriguez-Torres M. Patel K. (2013). Treatment of HCV infection by targeting microRNA. *N. Engl. J. Med.* 368 1685–1694 10.1056/NEJMoa1209026 23534542

[B29] Kogure T. Lin W. L. Yan I. K. Braconi C. Patel T. (2011). Intercellular nanovesicle-mediated microRNA transfer: a mechanism of environmental modulation of hepatocellular cancer cell growth. *Hepatology* 54 1237–1248 10.1002/hep.24504 21721029 PMC3310362

[B30] Kohler C. Barekati Z. Radpour R. Zhong X. Y. (2011). Cell-free DNA in the circulation as a potential cancer biomarker. *Anticancer Res.* 31 2623–2628 10.1186/1476-4598-8-105 21778314

[B31] Kosaka N. Iguchi H. Yoshioka Y. Takeshita F. Matsuki Y. Ochiya T. (2010). Secretory mechanisms and intercellular transfer of microRNAs in living cells. *J. Biol. Chem.* 285 17442–17452 10.1074/jbc.M110.107821 20353945 PMC2878508

[B32] Kuwabara Y. Ono K. Horie T. Nishi H. Nagao K. Kinoshita M. (2011). Increased microRNA-1 and microRNA-133a levels in serum of patients with cardiovascular disease indicate myocardial damage. *Circ. Cardiovasc. Genet.* 4 446–454 10.1161/CIRCGENETICS.110.958975 21642241

[B33] Laterza O. F. Lim L. Garrett-Engele P. W. Vlasakova K. Muniappa N. Tanaka W. K. (2009). Plasma microRNAs as sensitive and specific biomarkers of tissue injury. *Clin. Chem.* 55 1977–1983 10.1373/clinchem.2009.131797 19745058

[B34] Lawrie C. H. Gal S. Dunlop H. M. Pushkaran B. Liggins A. P. Pulford K. (2008). Detection of elevated levels of tumor-associated microRNAs in serum of patients with diffuse large B-cell lymphoma. *Br. J. Haematol.* 141 672–675 10.1111/j.1365-2141.2008.07077.x 18318758

[B35] Lee L. W. Zhang S. Etheridge A. Ma L. Martin D. Galas D. (2010). Complexity of the microRNA repertoire revealed by next-generation sequencing. *RNA* 16 2170–2180 10.1261/rna.2225110 20876832 PMC2957056

[B36] Lee S. T. Chu K. Jung K. H. Kim J. H. Huh J. Y. Yoon H. (2012). miR-206 regulates brain-derived neurotrophic factor in Alzheimer disease model. *Ann. Neurol.* 72 269–277 10.1002/ana.23588 22926857

[B37] Lim P. K. Bliss S. A. Patel S. A. Taborga M. Dave M. A. Gregory L. A. (2011). Gap junction-mediated import of microRNA from bone marrow stromal cells can elicit cell cycle quiescence in breast cancer cells. *Cancer Res.* 71 1550–1560 10.1158/0008-5472.CAN-10-2372 21343399

[B38] Liu A. Y. Pascal L. E. Vencio R. Z. Vencio E. F. (2010). Stromal–epithelial interactions in early neoplasia. *Cancer Biomarker* 9 141–155 10.3233/CBM-2011-0174 PMC13015978 22112474

[B39] Lo Y. M. Chiu R. W. (2012). Genomic analysis of fetal nucleic acids in maternal blood. *Annu. Rev. Genomics Hum. Genet.* 13 285–306 10.1146/annurev-genom-090711-163806 22657389

[B40] Lotvall J. Valadi H. (2007). Cell to cell signalling via exosomes through esRNA. *Cell Adh. Migr.* 1 156–158 10.4161/cam.1.3.5114 19262134 PMC2634021

[B41] Ma Y. Zhang P. Wang F. Zhang H. Yang J. Peng J. (2012). miR-150 as a potential biomarker associated with prognosis and therapeutic outcome in colorectal cancer. *Gut* 61 1447–1453 10.1136/gutjnl-2011-301122 22052060

[B42] Mandel P. Métais P. (1948). Les acides nucléiques du plasma sanguin chez l’homme. *C. R. Acad. Sci. Paris* 142 241–243 10.1373/clinchem.2005.051003 18875018

[B43] Meckes D. G. >Jr. Shair K. H. Y. Marquitz A. R. Kung C. P. Edwards R. H. Raab-Traub N. (2010). Human tumor virus utilizes exosomes for intercellular communication. *Proc. Natl. Acad. Sci. U.S.A.* 107 20370–20375 10.1073/pnas.1014194107 21059916 PMC2996715

[B44] Meder B. Keller A. Vogel B. Haas J. Sedaghat-Hamedani F. Kayvanpour E. (2011). MicroRNA signatures in total peripheral blood as novel biomarkers for acute myocardial infarction. *Basic Res. Cardiol.* 106 13–23 10.1007/s00395-010-0123-2 20886220

[B45] Mitchell P. S. Parkin R. K. Kroh E. M. Fritz B. R. Wyman S. K. Pogosova-Agadjanyan E. L. (2008). Circulating microRNAs as stable blood-based markers for cancer detection. *Proc. Natl. Acad. Sci. U.S.A.* 105 10513–10518 10.1073/pnas.0804549105 18663219 PMC2492472

[B46] Mittelbrunn M. Gutierrez-Vazquez C. Villarroya-Celtri C. Gonzalez S. Sanchez-Cabo F. Gonzalez M. A. (2011). Unidirectional transfer of microRNA-loaded exosomes from T cells to antigen-presenting cells. *Nat. Commun.* 2 282 10.1038/ncomms1285 PMC3104548 21505438

[B47] Miyamoto M. Yanai M. Ookubo S. Awasaki N. Takami K. Imai R. (2008). Detection of cell-free, liver-specific mRNAs in peripheral blood from rats with hepatotoxicity: a potential toxicological biomarker for safety evaluation. *Toxicol. Sci.* 106 538–545 10.1093/toxsci/kfn188 18779383

[B48] Mizuno H. Nakamura A. Aoki Y. Ito N. Kishi S. Yamamoto K. (2011). Identification of muscle-specific microRNAs in serum of muscular dystrophy animal models: promising novel blood-based markers for muscular dystrophy. *PLoS ONE* 6: e18388 10.1371/journal.pone.0018388 PMC3068182 21479190

[B49] Montecalvo A. Larregina A. T. Shufesky W. J. Stolz D. B. Sullivan M. L. Karlsson J. M. (2012). Mechanism of transfer of functional microRNAs between mouse dendritic cells via exosomes. *Blood* 119 756–766 10.1182/blood-2011-02-338004 22031862 PMC3265200

[B50] Moussay E. Wang K. Cho J. H. van Moer K. Pierson S. Paggetti J. (2011). MicroRNA as biomarkers and regulators in B-cell chronic lymphocytic leukemia. *Proc. Natl. Acad. Sci. U.S.A.* 108 6573–6578 10.1073/pnas.1019557108 21460253 PMC3081030

[B51] Muller G. (2012). Microvesicles/exosomes as potential novel biomarkers of metabolic diseases. *Diabetes Metab. Syndr. Obes.* 5 247–282 10.2147/DMSO.S32923 22924003 PMC3422911

[B52] Ohshima K. Inoue K. Fujiwara A. Hatakeyama K. Kanto K. Watanabe Y. (2010). Let-7 microRNA family is selectively secreted into the extracellular environment via exosomes in a metastatic gastric cancer cell line. *PLoS ONE* 5: e13247 10.1371/journal.pone.0013247 PMC2951912 20949044

[B53] Okubo S. Miyamoto M. Takami K. Kanki M. Ono A. Nakatsu N. (2013). Identification of novel liver-specific mRNAs in plasma for biomarkers of drug-induced liver injury and quantitative evaluation in rats treated with various hepatotoxic compounds. *Toxicol. Sci.* 132 21–31 10.1093/toxsci/kfs340 23288050

[B54] Park N. J. Zhou H. Elashoff D. Henson B. S. Kastratovic D. A. Abemayor E. (2009). Salivary microRNA: discovery, characterization, and clinical utility for oral cancer detection. *Clin. Cancer Res.* 15 5473–5477 10.1158/1078-0432.CCR-09-0736 19706812 PMC2752355

[B55] Pegtel D. M. Cosmopoulos K. Thorley-Lawson D. A. van Eijndhoven M. A. Hopmans E. S. Lindenberg J. L. (2010). Functional delivery of viral miRNAs via exosomes. *Proc. Natl. Acad. Sci. U.S.A.* 107 6328–6333 10.1073/pnas.0914843107 20304794 PMC2851954

[B56] Quinn S. R O’Neill L. A. (2011). A trio of microRNAs that control toll-like receptor signaling. *Int. Immunol.* 23 421–425 10.1093/intimm/dxr034 21652514

[B57] Rajakumar A. Cerdeira A. S. Rana S. Zsengeller Z. Edmunds L. Jeyabalan A. (2012). Transcriptionally active syncytial aggregates in the maternal circulation may contribute to circulating soluble fms-like tyrosine kinase 1 in preeclampsia. *Hypertension* 59 256–264 10.1161/HYPERTENSIONAHA.111.182170 22215706 PMC3319764

[B58] Ratajczak J. Miekus K. Kucia M. Zhang J. Reca R. Dvorak P. (2006). Embryonic stem cell-derived microvesicles reprogram hematopoietic progenitors: evidence for horizontal transfer of mRNA and protein delivery. *Leukemia* 20 847–856 10.1038/sj.leu.2404132 16453000

[B59] Roth P. Wischhusen J. Happold C. Chandran P. A. Hofer S. Eisele G. (2011). A specific miRNA signature in the peripheral blood of glioblastoma patients. *J. Neurochem.* 118 449–457 10.1111/j.1471-4159.2011.07307.x 21561454

[B60] Sayed D. Rane S. Lypowy J. He M. Chen I. Y. Vashistha H. (2008). MicroRNA-21 targets Sprouty2 and promotes cellular outgrowths. *Mol. Biol. Cell* 19 3272–3282 10.1091/mbc.E08-02-0159 18508928 PMC2488276

[B61] Schmidt W. M. Spiel A. O. Jilma B. Wolzt M. Muller M. (2009). In vivo profile of the human leukocyte microRNA response to endotoxemia. *Biochem. Biophys. Res. Commun.* 380 437–441 10.1016/j.bbrc.2008.12.190 19284987

[B62] Schonbeck U. Libby P. (2001). The CD40/CD154 receptor/ligand dyad. *Cell. Mol. Life Sci.* 58 4–43 10.1007/PL00000776 11229815 PMC11146501

[B63] Selvamani A. Sathyan P. Miranda R. C. Sohrabji F. (2012). An antagomir to microRNA Let7f promotes neuroprotection in an ischemic stroke model. *PLoS ONE* 7: e32662 10.1371/journal.pone.0032662 PMC3290559 22393433

[B64] Semenov D. V. Baryakin D. N. Brenner E. V. Kurilshikov A. M. Vasiliev G. V. Bryzgalov L. A. (2012). Unbiased approach to profile the variety of small non-coding RNA of human blood plasma with massively parallel sequencing technology. *Expert Opin. Biol. Ther.* 12(Suppl. 1) S43–S51 10.1517/14712598.2012.679653 22509727

[B65] Sheedy F. J O’Neill L. A. (2008). Adding fuel to fire: microRNAs as a new class of mediators of inflammation. *Ann. Rheum. Dis.* 67(Suppl. 3) iii50–iii55 10.1136/ard.2008.100289 19022814

[B66] Shi B. Gao W. Wang J. (2012). Sequence fingerprints of microRNA conservation. *PLoS ONE* 7: e48256 10.1371/journal.pone.0048256 PMC3480475 23110219

[B67] Skog J. Wurdinger T. van Rijn S. Meijer D. H. Gainche L. Sena-Esteves M. (2008). Glioblastoma microvesicles transport RNA and proteins that promote tumour growth and provide diagnostic biomarkers. *Nat. Cell Biol.* 10 1470–1476 10.1038/ncb1800 19011622 PMC3423894

[B68] Sonkoly E. Stahle M. Pivarcsi A. (2008). MicroRNAs and immunity: novel players in the regulation of normal immune function and inflammation. *Semin. Cancer Biol.* 18 131–140 10.1016/j.semcancer.2008.01.005 18291670

[B69] Starkey Lewis P. J. Dear J. Platt V. Simpson K. J. Craig D. G. Antoine D. J. (2011). Circulating microRNAs as potential markers of human drug-induced liver injury. *Hepatology* 54 1767–1776 10.1002/hep.24538 22045675

[B70] Su Y. W. Chen X. Jiang Z. Z. Wang T. Wang C. Zhang Y. (2012). A panel of serum microRNAs as specific biomarkers for diagnosis of compound- and herb-induced liver injury in rats. *PLoS ONE* 7: e37395 10.1371/journal.pone.0037395 PMC3356255 22624025

[B71] Suckau L. Fechner H. Chemaly E. Krohn S. Hadri L. Kockskamper J. (2009). Long-term cardiac-targeted RNA interference for the treatment of heart failure restores cardiac function and reduces pathological hypertrophy. *Circulation* 119 1241–1252 10.1161/CIRCULATIONAHA.108.783852 19237664 PMC4298485

[B72] Sueoka E. Sueoka N. Iwanaga K. Sato A. Suga K. Hayashi S. (2005). Detection of plasma hnRNP B1 mRNA, a new cancer biomarker, in lung cancer patients by quantitative real-time polymerase chain reaction. *Lung Cancer* 48 77–83 10.1016/j.lungcan.2004.10.007 15777973

[B73] Taylor D. D. Gercel-Taylor C. (2008). MicroRNA signatures of tumor-derived exosomes as diagnostic biomarkers of ovarian cancer. *Gynecol. Oncol.* 110 13–21 10.1016/j.ygyno.2008.04.033 18589210

[B74] Tijsen A. J. Creemers E. E. Moerland P. D. de Windt L. J. van der Wal A. C. Kok W. E. (2010). MiR423-5p as a circulating biomarker for heart failure. *Circ. Res.* 106 1035–1039 10.1161/CIRCRESAHA.110.218297 20185794

[B75] Turchinovich A. Burwinkel B. (2012). Distinct AGO1 and AGO2 associated miRNA profiles in human cells and blood plasma. *RNA Biol.* 9 1066–1075 10.4161/rna.21083 22858679 PMC3551861

[B76] Turchinovich A. Weiz L. Langheinz A. Burwinkel B. (2011). Characterization of extracellular circulating microRNA. *Nucleic Acids Res.* 39 7223–7233 10.1093/nar/gkr254 21609964 PMC3167594

[B77] Valadi H. Ekstrom K. Bossios A. Sjostrand M. Lee J. J. Lotvall J. O. (2007). Exosome-mediated transfer of mRNAs and microRNAs is a novel mechanism of genetic exchange between cells. *Nat. Cell Biol.* 9 654–659 10.1038/ncb1596 17486113

[B78] Valiunas V. Polosina Y. Y. Miller H. Potapova I. A. Valiuniene L. Doronin S. (2005). Connexin-specific cell-to-cell transfer of short interfering RNA by gap junctions. *J. Physiol.* 568 459–468 10.1113/jphysiol.2005.090985 16037090 PMC1474730

[B79] van Solingen C. Seghers L. Bijkerk R. Duijs J. M. Roeten M. K. van Oeveren-Rietdijk A. M. (2009). Antagomir-mediated silencing of endothelial cell specific microRNA-126 impairs ischemia-induced angiogenesis. *J. Cell. Mol. Med.* 13 1577–1585 10.1111/j.1582-4934.2008.00613.x 19120690 PMC3828868

[B80] Vencio E. F. Pascal L. E. Page L. S. Denyer G. Wang A. J. Ruohola-Baker H. (2011). Embryonal carcinoma cell induction of miRNA and mRNA changes in co-cultured prostate stromal fibromuscular cells. *J. Cell. Physiol.* 226 1479–1488 10.1002/jcp.22464 20945389 PMC3968429

[B81] Vickers K. C. Palmisano B. T. Shoucri B. M. Shamburek R. D. Remaley A. T. (2011). MicroRNAs are transported in plasma and delivered to recipient cells by high-density lipoproteins. *Nat. Cell Biol.* 13 423–433 10.1038/ncb2210 21423178 PMC3074610

[B82] Vignier N. Amor F. Fogel P. Duvallet A. Poupiot J. Charrier S. (2013). Distinctive serum miRNA profile in mouse models of striated muscular pathologies. *PLoS ONE* >8: e55281 10.1371/journal.pone.0055281 PMC3572119 23418438

[B83] Wagner J. Riwanto M. Besler C. Knau A. Fichtlscherer S. Roxe T. (2013). Characterization of levels and cellular transfer of circulating lipoprotein-bound microRNAs. *Arterioscler. Thromb. Vasc. Biol*. 33 1392–1400 10.1161/ATVBAHA.112.300741 23559634

[B84] Wang G. K. Zhu J. Q. Zhang J. T. Li Q. Li Y. He J. (2010a). Circulating microRNA: a novel potential biomarker for early diagnosis of acute myocardial infarction in humans. *Eur. Heart J.* 31 659–666 10.1093/eurheartj/ehq013 20159880

[B85] Wang K. Zhang S. Weber J. Baxter D. Galas D. J. (2010b). Export of microRNAs and microRNA-protective protein by mammalian cells. *Nucleic Acids Res.* 38 7248–7259 10.1093/nar/gkq601 20615901 PMC2978372

[B86] Wang K. Li H. Yuan Y. Etheridge A. Zhou Y. Huang D. (2012). The complex exogenous RNA spectra in human plasma: an interface with human gut biota? *PLoS ONE* 7: e51009 10.1371/journal.pone.0051009 PMC3519536 23251414

[B87] Wang K. Yuan Y. Li H. Cho J. H. Huang D. Gray L. (2013). The spectrum of circulating RNA, a window into systems toxicology. *Toxicol. Sci* 132 478–492 10.1093/toxsci/kft014 23358195

[B88] Wang K. Zhang S. Marzolf B. Troisch P. Brightman A. Hu Z. (2009). Circulating microRNAs, potential biomarkers for drug-induced liver injury. *Proc. Natl. Acad. Sci. U.S.A.* 106 4402–4407 10.1073/pnas.0813371106 19246379 PMC2657429

[B89] Wang R. Li N. Zhang Y. Ran Y. Pu J. (2011). Circulating microRNAs are promising novel biomarkers of acute myocardial infarction. *Intern. Med.* 50 1789–1795 10.2169/internalmedicine.50.5129 21881276

[B90] Weber J. A. Baxter D. H. Zhang S. Huang D. Y. Huang K. H. Lee M. J. (2010). The microRNA spectrum in 12 body fluids. *Clin. Chem.* 56 1733–1741 10.1373/clinchem.2010.147405 20847327 PMC4846276

[B91] Wolvetang E. J. Pera M. F. Zuckerman K. S. (2007). Gap junction mediated transport of shRNA between human embryonic stem cells. *Biochem. Biophys. Res. Commun.* 363 610–615 10.1016/j.bbrc.2007.09.035 17900528

[B92] Wu L. Zhou H. Lin H. Qi J. Zhu C. Gao Z. (2012). Circulating microRNAs are elevated in plasma from severe preeclamptic pregnancies. *Reproduction* 143 389–397 10.1530/REP-11-0304 22187671

[B93] Yang M. Chen J. Su F. Yu B. Lin L. Liu Y. (2011). Microvesicles secreted by macrophages shuttle invasion-potentiating microRNAs into breast cancer cells. *Mol. Cancer* 10 117 10.1186/1476-4598-10-117 PMC3190352 21939504

[B94] Yao Q. Xu H. Zhang Q. Q. Zhou H. Qu L. H. (2009). MicroRNA-21 promotes cell proliferation and down-regulates the expression of programmed cell death 4 (PDCD4) in HeLa cervical carcinoma cells. *Biochem. Biophys. Res. Commun.* 388 539–542 10.1016/j.bbrc.2009.08.044 19682430

[B95] Zhang L. Hou D. Chen X. Li D. Zhu L. Zhang Y. (2012a). Exogenous plant MIR168a specifically targets mammalian LDLRAP1: evidence of cross-kingdom regulation by microRNA. *Cell Res.* 22 107–126 10.1038/cr.2011.158 21931358 PMC3351925

[B96] Zhang X. Wang C. Wang L. Du L. Wang S. Zheng G. (2012b). Detection of circulating Bmi-1 mRNA in plasma and its potential diagnostic and prognostic value for uterine cervical cancer. *Int. J. Cancer* 131 165–172 10.1002/ijc.26360 21858805

[B97] Zhang Y. Liu D. Chen X. Li J. Li L. Bian Z. (2010). Secreted monocytic miR-150 enhances targeted endothelial cell migration. *Mol. Cell* 39 133–144 10.1016/j.molcel.2010.06.010 20603081

[B98] Zhao H. Shen J. Medico L. Wang D. Ambrosone C. B. Liu S. (2010). A pilot study of circulating miRNAs as potential biomarkers of early stage breast cancer. *PLoS ONE* 5: >e13735 10.1371/journal.pone.0013735 PMC2966402 21060830

[B99] Zhao Z. Zhao Q. Warrick J. Lockwood C. M. Woodworth A. Moley K. H. (2012). Circulating microRNA miR-323-3p as a biomarker of ectopic pregnancy. *Clin. Chem.* 58 896–905 10.1373/clinchem.2011.179283 22395025 PMC3694411

[B100] Zhong S. Zhang S. Bair E. Nares S. Khan A. A. (2012). Differential expression of microRNAs in normal and inflamed human pulps. *J. Endod.* 38 746–752 10.1016/j.joen.2012.02.020 22595106

[B101] Zhong X. Y. Holzgreve W. Huang D. J. (2008). Isolation of cell-free RNA from maternal plasma. *Methods Mol. Biol.* 444 269–273 10.1007/978-1-59745-066-9_21 18425488

